# Shrinking Lung Syndrome: A Rare Manifestation of Systemic Lupus Erythematosus

**DOI:** 10.7759/cureus.8216

**Published:** 2020-05-21

**Authors:** Saiara Choudhury, Manuel Ramos, Humayun Anjum, Mohammed Ali, Salim Surani

**Affiliations:** 1 Internal Medicine, Corpus Christi Medical Center, Corpus Christi, USA; 2 Pulmonary Medicine, Corpus Christi Medical Center, Corpus Christi, USA; 3 Pulmonary/Critical Care Medicine, Corpus Christi Medical Center, Corpus Christi, USA; 4 Internal Medicine, University of North Texas, Dallas, USA

**Keywords:** shrinking lung syndrome, systemic lupus erythematosus, dyspnea, restrictive lung disease, hypoxemia, diaphragmatic elevation

## Abstract

Shrinking lung syndrome (SLS) is a pulmonary complication of systemic lupus erythematosus (SLE) characterized by dyspnea, pleuritic chest pain, and progressive decrease in lung volumes with no evidence of pleural or interstitial disease on chest CT. We present a 51-year-old female with a 14-year history of SLE with symptoms of progressive shortness of breath, pleuritic chest pains, low grade fevers, and productive cough which was unresponsive to multiple courses of antibiotics. After careful review of her course of SLE and timeline of symptoms, she was diagnosed with SLS. Even though rare, clinicians should have a high suspicion of SLS in patients with a long-term history of SLE and worsening dyspnea. Early treatment can be initiated to help reduce long-term morbidity and mortality and maintain the quality of life.

## Introduction

Shrinking lung syndrome (SLS) is a rare pulmonary complication of systemic lupus erythematosus (SLE). It is characterized by progressive dyspnea, elevation of diaphragm, pleuritic chest pain, decreased lung volumes on imaging, and restrictive pattern seen in the pulmonary function tests (PFTs) [1]. Prevalence of SLS is estimated to be between 0.5% and 1.1% among patients with SLE [2]. Its pathophysiology remains largely unclear; however, hypotheses have been suggested ranging from microatelectatic changes due to lack of surfactant and increased surface tension, diaphragm fibrosis, and phrenic nerve palsy [3]. There is no standardized treatment of SLS, though most of these patients are treated with medium to high dose steroids [1]. Monoclonal antibodies to B-lymphocyte antigen CD (cluster of differentiation) 20 are also being studied as a possible treatment [4]. Despite lack of standardized and available therapy, the overall mortality seems to be low [1].

## Case presentation

A 51-year-old Hispanic female with the past medical history significant for the diagnosis of SLE, hypothyroidism, obesity and diabetes mellitus, presented to our pulmonary clinic for persistent shortness of breath worsening over past several months. She was seen in the remote past for shortness of breath, however, did not follow up for several years.

**Figure 1:**
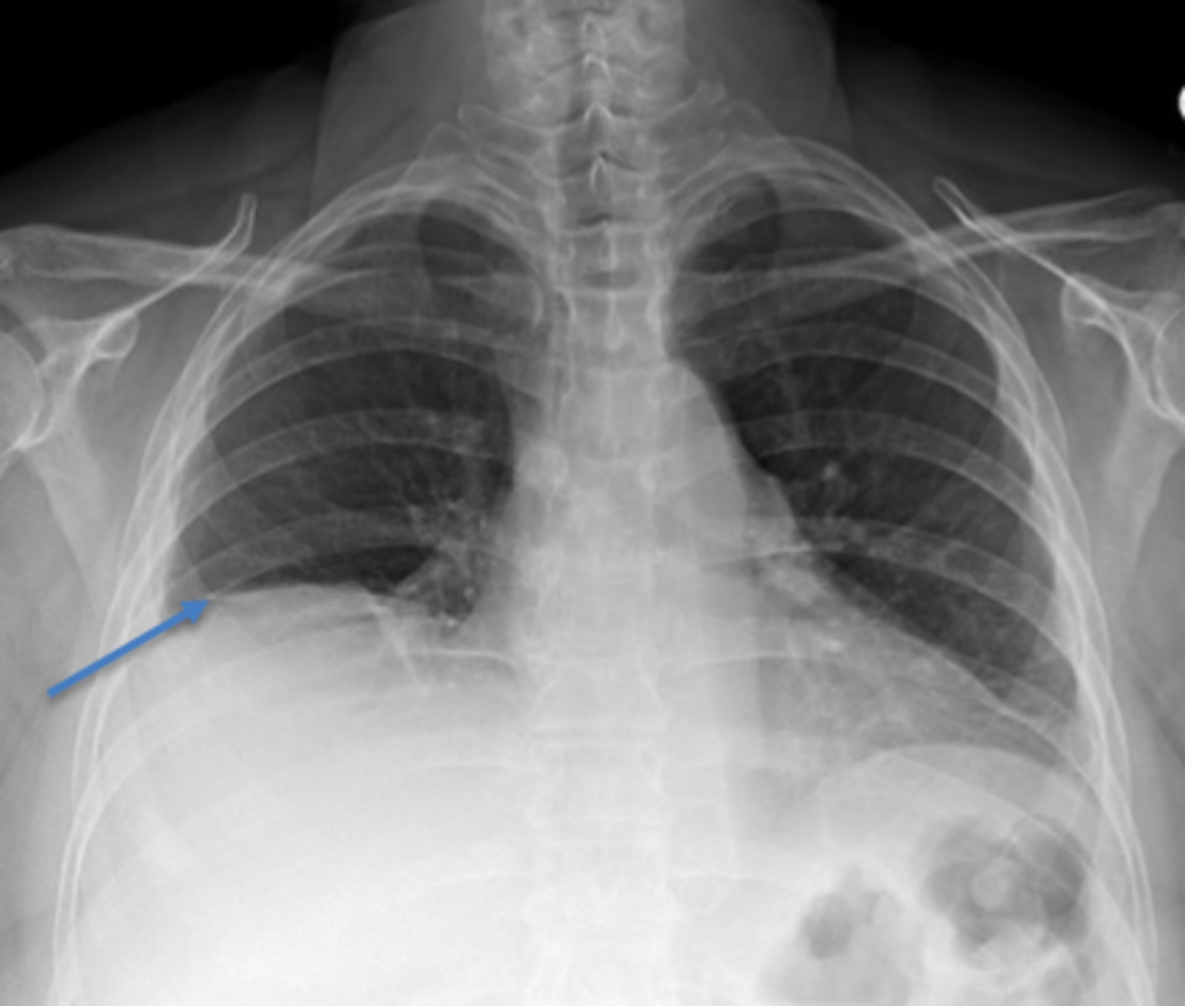
Chest X-ray showing low lung volume, elevated right hemidiaphragm, and normal pulmonary parenchyma.

**Figure 2:**
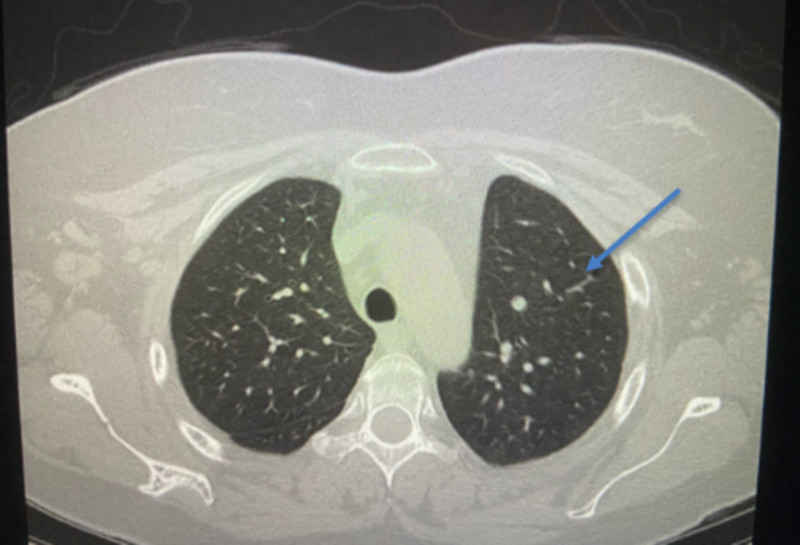
CT scan of chest without contrast revealing normal pulmonary parenchyma.

Some 15 years prior to this presentation in 2003, she had presented with the complaints of right-sided pleuritic chest pain, mild shortness of breath, and skin involvement. She was being followed intermittently by a rheumatologist for nonpulmonary manifestations of SLE and continued to have flare-ups. In December 2018, she started having a productive cough, worsening shortness of breath, low grade fever, and worsening skin involvement. She underwent three courses of different antibiotics, but her symptoms persisted. Upon evaluation in the clinic, it was noted that her pulmonary symptoms had been progressively getting worse. She had undergone multiple prior PFTs in 2007, 2008, and 2009 which showed a restrictive pattern with her lowest total lung capacity (TLC) being 49% of predicted (PP), lowest forced expiratory volume in one second (FEV1) being 33%, of predicted, and lowest diffusion capacity of the lungs for carbon monoxide (DLCO) being 9% of predicted. On this visit, repeat PFTs demonstrated TLC of 38% of predicted, FEV1 26%, and DLCO 36%. Table 1 shows PFTs in 2009 compared to PFTs in 2019. The patient underwent chest X-ray which showed smaller lung volume with raised right hemidiaphragm (Figure 1). The patient also underwent CT scan of chest which showed normal lung parenchyma with no pleural effusion (Figure 2).

**Table 1:** Pulmonary function test showing severe restrictive pattern with decreased FVC, decreased FEV1, increased FEV1/FVC, and decreased TLC. FVC, forced vital capacity; FEV, forced expiratory volume; TLC, total lung capacity; RV, residual volume; DLCO, diffusion capacity of the lungs for carbon monoxide

Spirometry	Predicted	Actual (2009)	% Predicted (2009)	Actual (2019)	% Predicted (2019)
FVC (L)	2.73	0.84	31	0.63	23
FEV1 (L)	2.21	0.73	33	0.58	26
FEV1/FVC (%)	81	86		91	112
RV (L)	1.52	1.2	79	0.80	52
TLC (L)	4.15	2.03	49	1.59	38
DLCO (mL/min/mmHg)	16.18	1.45	9	5.87	36

Flow volume loop performed in 2019 showed restrictive pattern.

Given her history of progressive SLE with worsening shortness of breath and progressive restrictive lung pattern, diagnosis of SLS was established. The patient was started on hydroxychloroquine and azathioprine. The patient’s symptoms continued to decline and azathioprine was discontinued and she was started on belimumab. The patient showed improvement in her symptoms and her dyspnea on exertion was improved. Follow up PFT is to be pending at this time (to be done six months post treatment).

## Discussion

Shrinking lung syndrome is still rarely described in medical literature with only 100 reported cases so far giving an estimated prevalence of <1% [5]. Majority of the cases are associated with dyspnea, decrease in lung volume with restrictive pattern, and elevation of hemidiaphragm. The greatest challenge in the diagnosis of SLS is the absence of other pulmonary pathologies such as interstitial, alveolar, or pulmonary disease [1, 6]. Our patient had the long-standing history of SLE with multiple co-morbidities including medical noncompliance and poor follow up. Her multiple flare-ups with shortness of breath, pleuritic chest pain, and productive cough could be associated with some common etiologies including pleuritis, pleural effusion, pulmonary vascular disease, or parenchymal disease but could also have been the early manifestations of her shrinking lung which is rare [6-7]. A case series by Ciaffi et al. highlights the challenges associated with diagnosis of SLS due to its rarity and because of it being diagnosis of exclusion [6]. Thus, increased awareness and suspicion is critical for early diagnosis and treatment. Early diagnosis and treatment can play an important role in preventing the disease progression and improving the morbidity and mortality. Our patient had the chronic and progressive complication of her SLE. Her PFTs have consistently shown a restrictive pattern with progressive decrease in the total lung capacity. Her multiple flare-ups requiring workup and treatment of antibiotics had failed to produce adequate and lasting relief of symptoms as well as satisfactory explanation for her pulmonary symptoms. Chest X-ray taken at the time of diagnosis showed an elevated right hemidiaphragm without any clear parenchymal disease. CT scan of the chest also showed normal pulmonary parenchyma with no pleural effusion or any evidence of any interstitial lung disease. This constellation of signs, symptoms, and diagnostic evidence led us to the diagnosis of SLS.

The lack of systematic review and research due to the rarity of reported cases continues to be a challenge in understanding of the pathophysiology and development of evidence-based management for this condition. Several mechanisms have been proposed as possible causes including respiratory myopathy, phrenic neuropathy, surfactant deficiency, and pleural adhesions [1, 7]. Omdal et al. presented a case of bilateral hemidiaphragm elevations and bibasilar atelectasis in a patient who originally had respiratory arrest. The patient was later diagnosed with SLE and shown to have phrenic neuropathy with electromyography and nerve conduction studies. They have also reported diaphragmatic paralysis and myopathy in SLE patients. This patient was treated with cyclophosphamide and methylprednisolone with gradual recovery [8]. Similarly, in our patient, elevation of her right hemidiaphragm could be caused by phrenic neuropathy and diaphragmatic myopathy due to prolonged poorly controlled SLE. 

Besides elevation of right hemidiaphragm, the restrictive lung pattern in our patient could also be caused by pleuritis. Along with her progressive shortness of breath, she also reported pleuritic chest pain on presentation. It has been shown that function of respiratory musculature is regulated partly by neuronal reflex arcs including intercostal-phrenic and pleural-phrenic reflexes [9]. Animal models have suggested that pleural inflammation has been associated with activation of the pleural-phrenic reflex leading to limited chest expansion [9-10]. Chronic inflammatory status in our patient due to poorly controlled SLE most likely had caused progressive development of pleuritis which could have contributed to the severe restrictive pattern leading to SLS.

There are no clear guidelines for management of this condition. Most cases of SLS associated with SLE were treated with steroids with significant symptomatic and pulmonary improvement [11]. Other immunosuppressive therapies such as azathioprine, cyclophosphamide, and rituximab have been shown to have some success in the treatment of SLS [11]. A retrospective study by Robles-Perez et al. showed improvement in DLCO and delayed need for lung transplant in patients who were treated with rituximab. However, this study only had 18 patients and 6/18 patients had adverse effects of rituximab [12]. Belimumab is a human immunoglobulin G1λ monoclonal antibody which blocks the binding of soluble B lymphocyte stimulators to the B cells. It is a once weekly subcutaneous injection and is the only biological agent currently approved for the treatment of nonrenal SLE [13]. Studies have shown that it is generally well tolerated as an add on therapy for patients with SLE. Further studies investigating the safety profile of belimumab has shown that it has favorable treatment results for lupus nephritis and helps in steroid rescue therapy in patients with SLE [14-15]. However, studies have not assessed the role of belimumab in patients with SLS. Our patient had presented to an outpatient clinic in stable condition. Due to her diabetes, the patient would have been a poor candidate for prolonged steroid use. She was initially started on hydroxychloroquine and azathioprine for immune modulating effects and was eventually treated with belimumab for maintenance therapy by rheumatology with improvement. 

## Conclusions

Shrinking lung syndrome is a rare manifestation of SLE. It remains under recognized and masquerades a diagnostic challenge. Patients with SLE, with normal lung parenchyma, no evidence of pleural effusion, and decrease in total lung capacity on PFT and elevated diaphragm with small lungs on imaging studies should alert the clinicians to the possibility of SLS. Early diagnosis can help in improving the morbidity and mortality. Clinicians need to consider SLS in the differential diagnosis of the patients with SLE who remain symptomatic with declining lung function.
